# When and why patients drop out from benign thyroid nodules follow-up: a single centre experience

**DOI:** 10.1007/s12020-022-03256-9

**Published:** 2022-11-25

**Authors:** Ilenia Pirola, Mario Rotondi, Elena Di Lodovico, Letizia Chiara Pezzaioli, Barbara Agosti, Maurizio Castellano, Alberto Ferlin, Carlo Cappelli

**Affiliations:** 1 Department of Clinical and Experimental Sciences, SSD Medicina ad indirizzo Endocrino-metabolico, University of Brescia, ASST Spedali Civili di Brescia, 25123 Brescia, Italy; 2 Unit of Internal Medicine and Endocrinology, Laboratory for Endocrine Disruptors, Istituti Clinici Scientifici Maugeri IRCCS, 27100 Pavia, Italy; 3 Department of Medicine, Unit of Andrology and Reproductive Medicine, University of Padova, Padova, Italy

**Keywords:** Thyroid ultrasound, Thyroid nodule, Drop-out

## Abstract

**Purpose:**

Drop-out in clinical long-term follow-up is a general problem that is potentially harmful to patients. No data about patients that drop out from thyroid ultrasound follow-up is available literature. The aim of the present retrospective study was to evaluate the characteristics of patients that dropped out from ultrasound thyroid nodule follow-up.

**Patients and methods:**

We reviewed medical records of all consecutive patients who underwent a fine needle aspiration from January 2007 to March 2009 in our department. All the patients with benign nodule(s) were recommended annual ultrasounds; patients who had dropped out from follow-up were included and a telephone interview was obtained to evaluate the reasons for dropping out.

**Results:**

289/966 (30%) of patients with benign nodules dropped out during follow-up; 94% of them within the first 5 years. Phone interviews were obtained from 201/289 (70%) of the patients. In the 57% of cases, the main declared reason for dropping out was nodular dimension stability during the first 2-3 years; 8.7% of them had forgotten about the appointment; 6.4% of subjects claimed to check only serum TSH, and 3.2% stated that they would undergo an ultrasound only if the nodule(s) were symptomatic. Finally, 10.7% patients continued follow-up in other centres.

**Conclusion:**

we showed that a third of patients miss their thyroid ultrasound follow-ups, and that the major cause is the low perceived threat coming from the disease. As a certain amount of drop-out is inevitable, attempting to reinforce our patients’ awareness regarding their own health state is mandatory.

**Trial registration:**

Trial registration: no. 4084.

## Introduction

Thyroid nodules are a common clinical problem worldwide [1]. In addition, the number of incidental thyroid lesions detected on imaging examinations performed for different reasons is growing, probably as a consequence of the increasing use of imaging exams [2].

Fine needle aspiration cytology (FNA) is the gold standard exam to differentiate benign from malignant lesions; in fact, it is safe, cost-effective and can be performed in an outpatient setting [3]. Fortunately, more than 90% of detected nodules are clinically benign lesions [4]. In this case, clinicians recommend serial ultrasound exams in accordance with the most authoritative guidelines [5, 6]. The purpose of monitoring is to identify nodular growth, with the assumption that this variable is an indication of malignancy and suggestive of the false diagnosis of a benign nodule at first evaluation.

We have recently observed in a large group of patients that a small number of benign lesions showed evidence of nodule growth during a follow-up period of 10 years [7]. However, in the same observational study 289/1248 patients (23%) dropped out during follow-up [7].

Drop-out in clinical long-term follow-up is a general problem that is potentially harmful to patients [8]. To the best of our knowledge, no data about patients that drop out from thyroid ultrasound follow-up have been reported in the available literature.

The aim of the present observational study was to evaluate the characteristics of patients that dropped out from ultrasound thyroid nodule follow-up.

## Subjects and methods

We reviewed the medical and imaging records of patients admitted to the FNA from January 2007 to March 2009. The indication to FNA was in accordance with the most authoritative guidelines edited in 2006 [9, 10].

All the patients with benign nodule(s) underwent annual ultrasound evaluations in our department. We used a LOGIQ 9 (GE, Healthcare, Milwaukee, WI, USA) or AplioTM500 (Toshiba Medical Systems Corp, Otawara, Japan) ultrasonographic scanner fitted with a 10–14-MHz linear transducer for morphological study.

We selected only patients who had dropped out from follow-up. Drop-out was considered for all patients who did not show up at annual ultrasound follow-up after the benign cytology report. A telephone interview was conducted in order to evaluate the reasons for dropping out.

The study was conducted according to the principles of the Helsinki Declaration and the guidelines of the Institutional Ethical Committee. All patients gave written consent for the storage and use of their data.

The study was approved by the Comitato Etico di Brescia (no. 4084).

## Statistical analysis

All data were collected in an electronic case report database. Statistical analyses were performed using SPSS 20.0 software (SPSS, Inc., Evanston, IL, USA).

Comparisons between groups and differences between proportions were calculated using *χ*^2^ for categorical variables and ANOVA test for quantitative variables, as appropriate. The Kaplan-Meier curve was fitted to determine the dropout time. Two-tailed *p* < 0.05 was considered statistically significant.

## Results

From January 2007 to March 2009, 1248 patients underwent thyroid fine needle aspiration cytology in our department. Among them, 966 (77.4%) patients were given a cytological diagnosis of benign nodule. During follow-up, 289/966 (30%) patients (202 women and 87 men), with a mean age of 52.5 (21–72) years dropped out.

The baseline features of the study population, comparing patients who did drop-out towards those who did not, are reported in Table 1. Patients were comparable for gender, TSH values and nodule characteristics. *In detail, hypoechoic pattern, blurred margins, microcalcifications and intranodular vascular pattern were fewer (but not significant) sings of malignancy of the drop-out group*. Differently, they were older (52.5 ± 14.3 vs 45.6 ± 13.9 yrs, *p* < 0.0001, respectively), and showed a higher number of multiple nodules than those who were retained (52.2 vs 44.8%, *p* = 0.019, respectively).

**Table 1:** Baseline characteristics of the study population, comparing patients who did drop-out towards those who did not

	Patients who did not drop-out	Patients who dropped-out	*p* Value
Gender (F/M)	474/203	202/87	0.514
Mean (SD) age	45.6 (13.9)	52.5 (14.3)	0.0001
Mean (SD) TSH, mIU/L	2.4 (0.8)	2.5 (0.7)	0.814
*Nodule Characteristics*			
No of multiple nodules (%)	303 (44.8%)	151 (52.2%)	0.019
Mean nodule volume in ml	2.4 (1.2)	2.4 (1.0)	0.568
Mean maximum diameter in mm (SD)	27.5 (9.4)	27.1 (8.8)	0.486
*Ultrasound findings*			
Hypoechoic pattern (%)	484 (71.5%)	197 (68.2%)	0.299
Blurred margins (%)	356 (52.5%)	106 (36.7%)	0.238
Microcalcifications (%)	428 (63.2%)	164 (56.7%)	0.269
Intranodular vascular pattern (%)	287 (42.3%)	121 (41.8%)	0.880

**Fig. 1:**
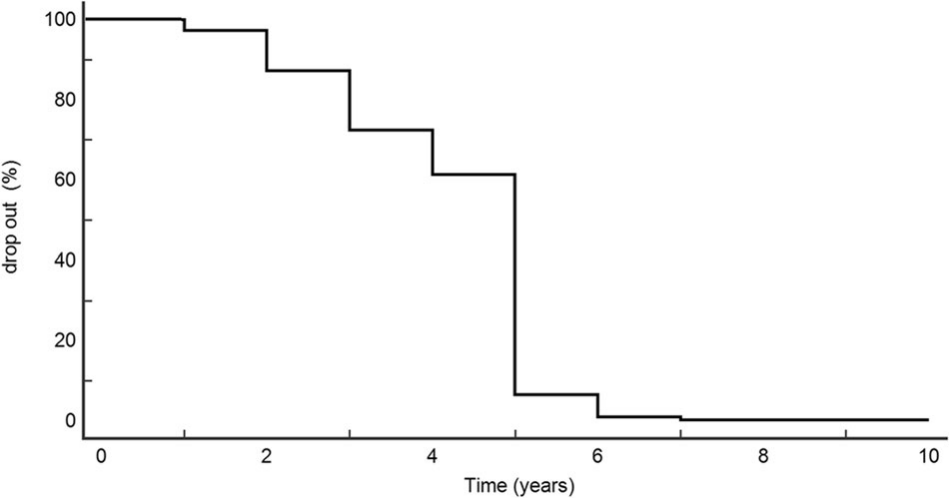
The Kaplan Meier dropout estimation among patients submitted to ultrasound follow-up

Significantly, 271/289 patients (94%) dropped out within the first 5 years of follow-up, as evidenced by the Kaplan–Meier curve, shown in Fig. 1. In detail, 2.8% of patients withdrew on the first appointment; 10, 14.9, and 11.1% of subjects dropped out at the second, third and fourth follow-up appointments, respectively. In the fifth year of follow-up, 55% of the patients did not show up for the scheduled check-up.

**Fig. 2:**
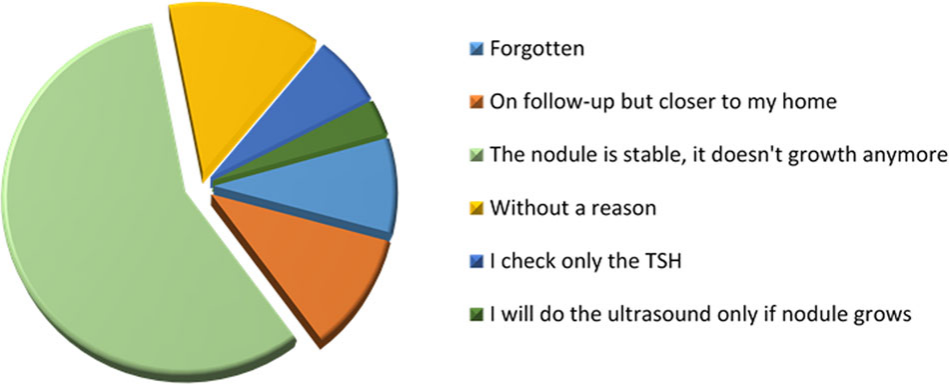
Reasons for missing scheduled clinic appointments

Phone interviews were obtained from 201/289 (70%) of the patients who were not retained, and the results are shown in Fig. 2.

In detail, 28/201 (14%) of patients did not provide a reason for dropping out, 8.7% had forgotten about the appointment, 13 (6.4%) subjects claimed to check only serum TSH, 3.2% stated that they would undergo an ultrasound evaluation only if the nodule(s) were bothering them, whereas 21 (10.7%) patients did proceed with follow-ups, but in a hospital closer to their home. In these last patients, no cases of malignancy were reported by phone interview.

Finally, 57% of the subjects declared that they dropped out from the follow-up as the nodule was stable during the first years, without any further growth. Among these patients, 89% dropped out at fifth years of follow-up.

## Discussion

This retrospective study showed that 30% of patients with benign thyroid nodules do not attend ultrasound follow-up. Moreover, 94% of them fail to attend within the first 5 years of monitoring.

### The American Thyroid Association (ATA) guidelines suggest ultrasound follow-up stratified accordingly to different ultrasound features, in patients with benign nodules

In particular, an ultrasound evaluation at 6–12 months for follow-up in high-risk ultrasound patterns, and no need in very low-risk group [5]. However, it is important to underline that these suggestions are based on an average-low quality of evidence, as reported by the ATA guidelines themselves [5]. Moreover, the American Association of Clinical Endocrinologists, American College of Endocrinology, and Associazione Medici Endocrinologi suggested to perform ultrasound examination in benign nodules in ~12 months after FNA. If nodules are unchanged repeat the exam after 24 months; this assumption was again based on low quality of evidence, as reported by the guidelines themselves [6].

In addition, a survey obtained from a large sample of patients referred to the Thyroid Nodule Clinic at the Brigham and Women’s Hospital in Boston, found that most benign thyroid nodule can be safely recommended for follow-up at 2–4 years with no risk of mortality or likelihood of harm [11]. Recently, our group showed that in a longer period of follow-up only 11% of patients with benign lesions showed evidence of nodule growth [7]. However, it is important to emphasise that the growth occurred in a linear fashion, starting from the first year of observation, and more important nodule growth started within the first 5 years of follow-up for 79% of the patients [7]. This is a key point, because in the present study we showed that almost all patients (94%) who dropped out of follow-up, were lost within the first 5 years of monitoring. Furthermore, the dimensional stability of the lesions within the first three years of follow-up was the main cause of dropout in the belief that the nodule (s) would no longer grow.

It is important to highlight that the purpose of monitoring is to identify nodular growth, considering this variable as an indicator of malignancy and suggesting a false diagnosis of a benign nodule at the first evaluation [12]. Indeed, how relevant is nodule growth as a marker of the development of malignancy is still matter of controversy [13].

Several factors related to patient, provider, disease, treatment, clinical administration, or environment, may affect clinic attendance. From orthodontic and orthopaedic surveys, it has been reported that clinical compliance decreases proportionally to the interval between the completion of treatment and the follow-up examination. The most common reasons for this include change of address, and death [8]. In diabetic patients, drop-out rates are quite high, ranging from 4 to 50% in different countries. The major reasons for this include low perceived concern for the disease and conflicts with work [14, 15]. To the best of our knowledge, no studies have specifically evaluated this topic in patients affected by thyroid diseases. In the present study, we showed that a large number (30%) of patients are lost during follow-up and we found that the majority of patients dropped out after a few checkups, in the first 5 years of monitoring, due to a ‘general’ low perceived concern for the disease (Fig. 2).

In agreement with Grover et al. [16], in our study drop-out was also associated with higher age, even if our population set was younger than those reported by colleagues. This result was also evidenced by a larger set of patients [17].

In few observation studies, a longer waiting time from referral to scheduled appointment was significantly associated with missed appointments [18].

Moreover, Murdock et al. reported forgetting an appointment, particularly in case of long waiting time, as the most common reason (36%) for nonattendance, in a survey-based study of gastroenterology patients [19]. Conversely, in our study less than 9% of subjects forgot their appointments. We have no explanation for this difference, as our institute has not adopted any reminder (i.e. letter, phone call or SMS) days before appointment, as was done in the above-mentioned study [19]. On the other hand, in our study, low perceived concern for the disease clearly emerged as the major cause of follow-up drop out. *We believe that standardised recordings of sonographic features, as EU-TIRADS* [20]*, will improve the selection of patients with potentially malignant nodules, who could be introduce in a recall system*.

In conclusion, we showed that a large set of patients miss their ultrasound thyroid follow-ups and a low perceived threat from the disease appears to be the major cause of this. As a certain amount of ‘reasons’ for patient drop-out are inevitable, attempting to reinforce our patients’ awareness regarding their own state of health is necessary.

## Data Availability

The data supporting the findings of this study are available from the corresponding author, upon request.
